# Physical Frailty: ICFSR International Clinical Practice Guidelines for Identification and Management

**DOI:** 10.1007/s12603-019-1273-z

**Published:** 2019-10-03

**Authors:** E. Dent, J. E. Morley, A. J. Cruz-Jentoft, L. Woodhouse, L. Rodríguez-Mañas, L. P. Fried, J. Woo, I. Aprahamian, A. Sanford, J. Lundy, F. Landi, J. Beilby, F. C. Martin, J. M. Bauer, L. Ferrucci, R. A. Merchant, B. Dong, H. Arai, E. O. Hoogendijk, C. W. Won, A. Abbatecola, T. Cederholm, T. Strandberg, L. M. Gutiérrez Robledo, L. Flicker, S. Bhasin, M. Aubertin-Leheudre, H. A. Bischoff-Ferrari, J. M. Guralnik, J. Muscedere, M. Pahor, J. Ruiz, A. M. Negm, J. Y. Reginster, D. L. Waters, B. Vellas

**Affiliations:** 1 Torrens University Australia, Adelaide, Australia; 2 Baker Heart and Diabetes Institute, Melbourne, Australia; 3 Division of Geriatric Medicine, Saint Louis University School of Medicine, St. Louis, Missouri USA; 4 Servicio de Geriatria, Hospital Universitario Ramón y Cajal (IRYCIS), Madrid, Spain; 5 Department of Physical Therapy, Rehabilitation Medicine, University of Alberta, Edmonton, Alberta Canada; 6 Servicio de Geriatria, Hospital Universitario de Getafe, Madrid, Spain; 7 Mailman School of Public Health, Columbia University Medical Center, New York, NY USA; 8 Department of Medicine, The Chinese University of Hong Kong, Hong Kong, China; 9 Geriatrics Division, Internal Medicine, Department, Faculty of Medicine of Jundiaí, Group of Investigation on Multimorbidity and Mental Health in Aging (GIMMA), Jundiaí, Brazil; 37 Medical School, University City of São Paulo, São Paulo, Brazil; 10 Perry County Memorial Hospital, Perryville, Missouri USA; 11 Fondazione Policlinico A. Gemelli, Roma, Italy; 12 Population Health Sciences, King’s College, London, UK; 13 Center for Geriatric Medicine, Heidelberg University Agaplesion Bethanien Krankenhaus, Heidelberg, Germany; 14 Intramural Research Program of the National Institute on Aging, Bethesda, USA; 15 Division of Geriatric Medicine, Department of Medicine, National University Hospital, National University Health System, Singapore, Singapore; 16 Department of Geriatrics and National Clinical Research Center for Geriatrics, West China Hospital of Sichuan University, Chengdu, China; 17 National Center for Geriatrics and Gerontology, Obu, Japan; 18 Department of Epidemiology and Biostatistics, Amsterdam Public Health Research institute, Amsterdam UMC — location VU University Medical Center, Amsterdam, the Netherlands; 19 Elderly Frailty Research Center, Department of Family Medicine, College of Medicine, Kyung Hee University, Seoul, Korea; 20 Alzheimer’s Disease Clinic Department, Azienda Sanitaria Locale (ASL) di Frosinone, Frosinone, Italy; 21 Clinical Nutrition and Metabolism, Department of Public Health and Caring Sciences, Uppsala University and Theme Aging, Karolinska University Hospital, Stockholm, Sweden; 22 Center for Life Course Health Research, University of Oulu, Oulu, Finland; 23 University of Helsinki and Helsinki University Hospital, Helsinki, Finland; 24 National Institute of Geriatrics, Mexico City, Mexico; 25 Western Australian Centre for Health and Ageing, Medical School, University of Western Australia, Perth, Australia; 26 Research Program in Mean’s Health: Aging and Metabolism, Boston Claude D. Pepper Older American Independence Center, Brigham and Women’s Hospital, Harvard Medical School, Boston, Massachusetts USA; 27 Dept des Sciences de l’activité physique, Université du Quebec à Montréal, CRIUGM, Montreal, Québec Canada; 28 Dept of Geriatrics and Aging Research, University Hospital and University of Zurich, Zurich, Switzerland; 29 Dept of Epidemiology and Public Health, Division of Gerontology, University of Maryland School of Medicine, Baltimore, Maryland USA; 30 Dept of Critical Care Medicine, Queen’s University, Kingston, Ontario Canada; 31 Dept of Aging and Geriatric Research, University of Florida, Gainesville, Florida USA; 32 Miami VA Healthcare System GRECC and Division of Geriatrics & Palliative Medicine, University of Miami Miller School of Medicine, Miami, Florida USA; 33 School of Rehabilitation Sciences, Faculty of Health Sciences, McMaster University, Hamilton, Ontario Canada; 34 WHO Collaborating Center for Public Health Aspects of Musculoskeletal Health and Aging, Liège, Belgium and Chair for Biomarkers of Chronic Diseases, Department of Biochemistry, College of Science, King Saud University, Riyadh, Kingdom of Saudi Arabia; 35 Dept of Medicine/School of Physiotherapy, University of Otago, Dunedin, New Zealand; 36 Gérontopôle UMR Inserm 1027, Université Paul Sabatier, CHU Toulouse, Toulouse, France

**Keywords:** Aged, 80 and over, Practice guideline, Frailty/diagnosis, Frailty/therapy*, Patient Care Planning/standards

## Abstract

**Objective:**

The task force of the International Conference of Frailty and Sarcopenia Research (ICFSR) developed these clinical practice guidelines to overview the current evidence-base and to provide recommendations for the identification and management of frailty in older adults.

**Methods:**

These recommendations were formed using the GRADE approach, which ranked the strength and certainty (quality) of the supporting evidence behind each recommendation. Where the evidence-base was limited or of low quality, Consensus Based Recommendations (CBRs) were formulated. The recommendations focus on the clinical and practical aspects of care for older people with frailty, and promote person-centred care.

**Recommendations for Screening and Assessment:**

The task force recommends that health practitioners case identify/screen all older adults for frailty using a validated instrument suitable for the specific setting or context (strong recommendation). Ideally, the screening instrument should exclude disability as part of the screening process. For individuals screened as positive for frailty, a more comprehensive clinical assessment should be performed to identify signs and underlying mechanisms of frailty (strong recommendation).

**Recommendations for Management:**

A comprehensive care plan for frailty should address polypharmacy (whether rational or nonrational), the management of sarcopenia, the treatable causes of weight loss, and the causes of exhaustion (depression, anaemia, hypotension, hypothyroidism, and B12 deficiency) (strong recommendation). All persons with frailty should receive social support as needed to address unmet needs and encourage adherence to a comprehensive care plan (strong recommendation). First-line therapy for the management of frailty should include a multi-component physical activity programme with a resistance-based training component (strong recommendation). Protein/caloric supplementation is recommended when weight loss or undernutrition are present (conditional recommendation). No recommendation was given for systematic additional therapies such as cognitive therapy, problem-solving therapy, vitamin D supplementation, and hormone-based treatment. Pharmacological treatment as presently available is not recommended therapy for the treatment of frailty.

**Electronic Supplementary Material:**

Supplementary material is available for this article at 10.1007/s12603-019-1273-z and is accessible for authorized users.

## Recommendations

**Table 1:** Summary of ICFSR evidence-based recommendations and clinical considerations for the identification and management of frailty in older adults

	**Recommendation**	**Grade**	**Certainty of Evidence**
*Frailty Screening*			
1	All adults aged 65 years and over should be offered screening for frailty using a validated rapid frailty instrument suitable to the specific setting or context	Strong	Low
*Frailty Assessment*			
2	Clinical assessment of frailty should be performed for all older adults screening as positive for frailty or pre-frailty	Strong	Low
*Development of a Comprehensive Management Plan*			
3	A comprehensive care plan for frailty should systematically address polypharmacy, the management of sarcopenia, treatable causes of weight loss, and the causes of fatigue (depression, anaemia, hypotension, hypothyroidism, and vitamin B12 deficiency)	Strong	Very Low
4	Where appropriate, persons with advanced (severe) frailty should be referred to a geriatrician	CBR	No data†
*Physical Activity/Exercise*			
5	Older people with frailty should be offered a multi-component physical activity programme (or those with pre-frailty as a preventative component)	Strong	Moderate
6	Health practitioners are strongly encouraged to refer older people with frailty to physical activity programmes with a progressive, resistance-training component	Strong	Moderate
*Nutrition and Oral Health*			
7	Protein/caloric supplementation can be considered for persons with frailty when weight loss or undernutrition has been diagnosed	Conditional	Very Low
8	Health practitioners may offer nutritional/protein supplementation paired with physical activity prescription	Conditional	Low
9	Advise older adults with frailty about the importance of oral health	CBR	No data†
*Pharmacological Intervention*			
10	Pharmacological treatment as presently available is not recommended therapy for the treatment of frailty	CBR	Very Low
*Additional Therapies and Treatments*			
11	Vitamin D supplementation is not recommended for the treatment of frailty unless vitamin D deficiency is present	CBR	Very low
12	Cognitive or problem-solving therapy is not systematically recommended for the treatment of frailty	CBR	Very low
13	Hormone therapy is not recommended for the treatment of frailty	CBR	Very low
14	All persons with frailty may be offered social support as needed to address unmet needs and encourage adherence to the Comprehensive Management Plan	Strong	Very low
15	Persons with frailty can be referred to home-based training	Conditional	Low

Where sufficient evidence was available from systematic reviews/meta-analyses, recommendations were ranked according to the GRADE approach (1). Where evidence was limited in systematic reviews/meta-analyses or for topics beyond the scope of systematic reviews, Consensus Based Recommendations (CBR) were formulated by the International Conference of Frailty and Sarcopenia Research (ICFSR) task force on frailty; † ‘No data’ indicates no data identified by systematic reviews.

## Introduction

Frailty is prevalent in all countries and is a leading contributor to functional decline and early mortality in older adults (2–4). The condition is defined as “a clinical state in which there is an increase in an individual’s vulnerability for developing an increased dependency and/or mortality when exposed to a stressor” (5). Frailty can begin before 65 years of age, but the onset escalates in those aged 70 years and over (6). Nonetheless, frailty is not an obligatory part of the ageing process, and many adults reach advanced ages without developing frailty (7). Accordingly, various long-term risk factors of frailty, like overweight/obesity (8, 9), physical inactivity (10), cardiovascular risk (11, 12), self-rated health (13), and alcohol use (14) have been identified.

The current estimate of physical frailty prevalence is around 15% for adults aged 65 years and over, based on a recent meta-analysis of community-dwelling older Europeans (15). In adults aged over 85 years, prevalence increases to over 25%. (16) Frailty prevalence is also elevated in persons with lower education, low socioeconomic position, or from ethnic minority groups (17–20). In addition, women tend to have a higher prevalence of frailty than men (6, 16, 20–23), although they may be more resistant to decline in frailty status over time (24). The number of older adults with frailty is increasing, likely due to increased survival of older adults with co-morbidities, more exposure to sedentary lifestyles, and smaller social support networks (25–27).

There is much potential for frailty to be reversed, particularly in its early stages (28–30). For that reason, early identification and management of frailty is an important priority for both healthcare providers and healthcare policy makers (31–33). This paper presents international Clinical Practice Guidelines (CPGs) for the identification and management of frailty.

### Guideline Objectives

These CPGs (hereafter known as “guidelines”) were developed to help ensure that all older people living with frailty are provided with high-quality, evidence-based care. The target audience for these guidelines includes all health professionals who contribute to the care of older people with frailty, including clinicians and allied health professionals; the target patient population includes all older adults with either frailty or at risk of frailty. These guidelines are not targeted towards those persons who already have well-established disability. The guidelines are applicable to both the hospital and primary care setting.

It is expected that implementation of these CPGs will improve both the short- and long-term outcomes of older adults with frailty. The guidelines aim to maintain or improve the physical function, health and experience of the care process for older people with frailty. Continuity of care is encouraged. An outline of current, relevant evidence will be provided regarding screening, assessment and management of frailty in older adults. Health practitioners will use these guidelines differently depending on their care setting and expertise. It is anticipated that national, state and local policy makers will be able to utilise these guidelines to develop health, social and aged care policies specific for older adults with frailty. Although these are international guidelines for frailty, the evidence-base comes predominantly from high-income countries.

## What is Physical Frailty?

**Figure 1:**
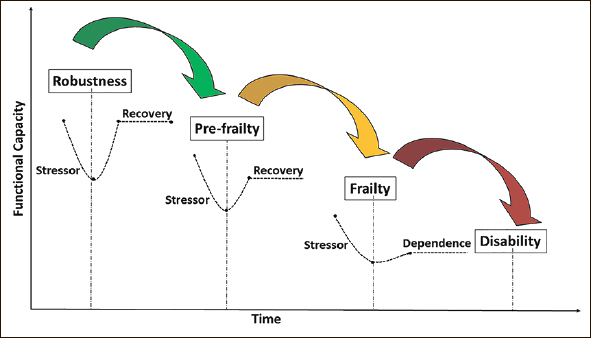
The cascade of functional decline in older adults from independence, through to frailty and disability (in the absence of intervention) [Based on concepts and findings by Dapp et al. (34) Hoogendijk et al. (35), Clegg et al. (36) and Fried et al. (37)]

Physical frailty can be considered as pre-disability, with disability defined as needing assistance with basic Activities of Daily Living (ADL). Figure 1 outlines the cascade of functional decline in older adults from independence through to frailty and disability. Targeted intervention can stall, slow or reverse this cascade of decline.

Frailty was first described by Fried and colleagues (37) in terms of its physical characteristics, or ‘phenotype’, and is objectively identified as three or more of five components: weakness (low grip strength), slowness (slow walking speed), shrinking (unintentional weight loss), exhaustion (self-reported), and low physical activity (37). Weight loss is usually the last of these five physical characteristics to manifest (38), and it is noted that once weight loss has occurred, it is very difficult to improve or reverse frailty status and physical functioning (39, 40). Understandably, the ‘anorexia of ageing’, which is age-related appetite and weight loss, is closely linked with the development of physical frailty (41–44).

Physical frailty is also related to sarcopenia [low muscle strength and/or muscle quantity of quality] (45–48), with clinical management of the two conditions overlapping somewhat (33, 49). Co-morbidity and disability also overlap with frailty, although they are both clinically and conceptually distinct (50, 51). For instance, among older people with frailty in the Montreal Unmet Needs Study (MUNS), 29.1% of people had an ADL disability and 81.8% had one or more co-morbidities. That is, although it is common for people with frailty to have disability or co-morbidity, a person can be frail without any co-morbidity or ADL disability, and vice versa. There is also a distinction between frailty and vulnerability, with frailty generally considered to be a severe state of vulnerability, where small perturbations in internal/external stressors can lead to functional decline (51).

Frailty is a dynamic entity where an individual can transition between states. For example, hospitalisation can transition an older adult from robust to frail (24, 52). On the other hand, being physically active may reverse frailty development — at least partially (29, 53, 54). Thus, understanding how an older person can dynamically move in and out of frailty states is important for both prevention and management of the condition.

## Guideline Development Process

These guidelines were formed by the International Conference of Frailty and Sarcopenia Research (ICFSR) which is an international alliance of subject-matter experts in the fields of both frailty and sarcopenia. An ICFSR expert working group on frailty (referred to as the ‘task force’ hereon) was formed, and included a multidisciplinary panel of practicing clinicians (predominantly geriatricians), Allied Health Professionals (AHPs), Primary Care Physicians (PCPs), musculoskeletal physiologists, gerontologists, and methodology experts. A steering committee was formed from the task force members, and was responsible for overseeing the development and review of the guideline development process. Input was also sought from four external reviewing groups: (i) two healthcare provider reviewing groups comprised of PCPs, therapists, geriatricians, nurse practitioners, residents in geriatric medicine, nurses specialising in geriatric medicine, and AHPs [Perry County, USA (n=7) and Madrid, Spain (n=15)]; and (ii) two healthcare consumer groups of older people with frailty [Perry County, USA (n=12) and Madrid, Spain (n=10)] (See Appendix).

Guidelines were developed using the Grading of Recommendations, Assessment, Development and Evaluation (GRADE) approach, which is an objective, structured system of ranking the strength and certainty (quality) of the supporting evidence behind each recommendation (guideline) (1). Where the evidence-base was limited or of low quality, Consensus Based Recommendations (CBRs) were formulated by the task force. These CBRs were voted on by task force members via an emailed survey, and a draft manuscript formed. The draft manuscript and included recommendations were then reviewed and discussed at the ICSFR task force workshop as part of the annual ICFSR conference (February 2019, Miami USA). Further iterations of the recommendations and manuscript were then made via email using an iterative Delphi process of expert consensus (55).

The recommendations focus on core practices for the identification and management of frailty in older adults and consider lifestyle factors as well as the clinical and practical aspects of care. We focus on physical frailty to enable us to divert from including comorbidity scales and psychosocial frailty. The management of multidimensional frailty and subtypes of frailty (cognitive, social and nutritional) are beyond scope. Although these guidelines do not specifically focus on the prevention of frailty, many recommendations are relevant to frailty prevention. A user-friendly patient information guide was also developed which was based on feedback from older adults with frailty and their caregivers (see Appendix).

### Searching the Evidence

These guidelines were informed by two systematic reviews which used the GRADE approach (56, 57), a systematic overview which also used the GRADE process (58), and other systematic reviews which included trials defining frailty using an objective, validated instrument (56, 58–63). In addition to the randomised clinical trials (RCTs) covered in these systematic reviews, relevant trials defining frailty objectively using a validated instrument were identified through comprehensive literature searches using PubMed and Scopus databases. Search term combinations included “frailty/diagnosis”, “frailty/therapy*”, “patient care planning/standards”, “geriatric assessment/statistics and numerical data”, “aged”, “intervention” and “treatment”. The libraries of task force members were also used to identify additional clinical trials on frailty treatment and management. Interventions focusing on community-dwelling older adults were prioritised to promote generalisability of the guidelines. Where RCTs were absent, other experimental designs such as observational studies were considered.

The Population, Intervention, Comparator and Outcomes (PICO) question used to inform the literature search query was:

For older adults with frailty or risk of frailty with or without co-morbid conditions and either residing in the community or currently admitted to hospital/a rehabilitation setting (P), what are the relative benefits and harms of different treatment/management strategies reported in randomised clinical trials (RCTs) (I) compared to usual care or placebo treatment (C) on change in physical frailty status or functioning between baseline and follow-up measured using a validated frailty instrument or a physical performance measure (O). Secondary outcomes were quality of life (QoL), cost effectiveness, physical performance measurement and any adverse clinical outcome. These primary and secondary outcomes were informed by a recent protocol paper (64).

### Grading the Strength and Certainty of Evidence: GRADE approach

The strength and certainty (quality) of evidence was evaluated for each recommendation as per the GRADE approach (1) where possible (See Table 2); specifically, this approach involves a formal assessment of the strength and certainty of evidence by outcome. The strength of a recommendation reflected the harm-benefit balance, patient values and preferences, cost-effectiveness and the quality of supporting evidence (65, 66). A strong recommendation indicated that the intervention was likely to have benefits that outweighed any risk, and that most clinicians would prescribe this intervention (65, 66). A conditional recommendation implied that whilst many clinicians would prescribe the intervention, many would not because of the fine balance between harms and benefits of the intervention (1, 67) (see Table 2). In addition, when grading the strength of each recommendation, the ICFSR task force strongly focused on two aspects from the GRADE Evidence-to-Decision (EtD) framework (1, 67): (i) person-centred outcomes important to older adults; and (ii) the number of older adults with frailty who would likely benefit from the intervention. Barriers to implementation of the guidelines (such as resources and access to services) were also considered by the task force when assigning grades to each recommendation.

**Table 2:** Categorical definitions for the strength and certainty of evidence, as per GRADE guidelines (1)

**Category**	**Description**
*Strength of the Recommendation*	
Strong	A strong recommendation indicates that the benefits of the intervention likely outweigh any associated risk; most clinicians would prescribe this intervention, and most patients would want to receive this type of intervention (65, 66).
Conditional (Weak)	A conditional recommendation indicates that clinicians would only refer the intervention under specific conditions because there is a fine balance between risks and burdens. Whilst many health practitioners would recommend the intervention, others would not; burdens include unwanted side effects and increased risk of adverse outcomes which undermine the health benefits of the intervention (57, 58). A conditional recommendation was also given when patient values were unknown regarding the intervention, or if there was substantial variation in patient preferences/values (1, 67)
*Certainty of Evidence†*	
High	Further research is very unlikely to change confidence in the estimate of effect.
Moderate	Further research is likely to have an important impact on our confidence in the estimate of effect and may change the estimate.
Low	Further research is very likely to have an important impact on our confidence in the estimate of effect and is likely to change the estimate.
Very Low	Any estimate of effect is very uncertain.

† As per the GRADE approach, the certainty of evidence was ranked lower by the task force when the following existed: study design limitation (including inconsistencies) and/or uncertainties (such as sparse or imprecise data, or indirect evidence); certainty of evidence was ranked higher when there was evidence of a dose-response gradient, no major threats to the validity of supporting studies, and/or consistent evidence with no confounding variables.

The certainty of evidence signified the overall certainty regarding the effectiveness of the intervention, and incorporated information regarding the trial design and number of participants, risk of bias, imprecision, inconsistency, indirectness and publication bias (1, 67). There were four gradings of certainty of evidence: high, moderate, low and very low (Table 2).

## Frailty Screening

### Recommendation 1: All adults aged 65 years and over should be offered opportunistic screening for frailty using a simple, validated frailty instrument suitable to the specific setting or context (Strong recommendation; low certainty of evidence)

The task force strongly recommends that older adults should be screened for frailty using a simple, validated frailty instrument suitable to the specific setting or context (task force agreement with recommendation: 78.9%). This recommendation also received unanimous support by the external reviewing group of healthcare providers, and strong support by both patient consumer groups.

#### Background: the rationale for frailty screening

The global burden of frailty has spurred an international effort to identify which instrument is most suited for frailty screening. An ideal screening tool needs to be effective not only in resource-limited areas, but also in developed countries where access to geriatric screening is often lacking. Frailty screening usually involves the recognition of functional decline alongside various other components which may or may not include slow gait speed, weight loss, cognitive difficulties, and exhaustion, depending on which screening tool is used.

Primary care appears to be the logical place to screen or case identify frailty in older persons (68, 69), particularly in its early stages when it is more likely to be amenable to intervention (28, 29, 31). However, many primary care practices (PCPs) are facing excessive patient loads, and must also divide their attention between diagnosing and managing chronic and infectious diseases that their older patients may present with. Further to this, many older adults have difficulties accessing health care services (70), which in turn is a major barrier to timely identification of frailty. Thus frailty should be identified in all healthcare encounters (33, 71).

#### Which screening instrument to use?

A frailty screening instrument needs to be efficient in identifying frailty (72). Currently, there exists a large assortment of frailty screening instruments, each with a variety of included components. Screening tools recommended by the ICFSR task force include Rockwood’s Clinical Frailty Scale (CFS) (73), the International Association of Nutrition and Ageing (IANA)’s FRAIL scale (74), and the Edmonton Frailty Scale (EFS) (75). The CFS is based on clinical judgement, and involves a nine-point pictorial scale paired with corresponding text describing classifications of frailty (73). At scores higher than 6, an individual can be considered as having a disability in ADL rather than frailty per se. Recently, the International Consortium for Health Outcomes Measurement (ICHOM) has recommended the CFS as part of its standard set of outcome measurements for studies of older adults (76). The FRAIL scale comprises five components: Fatigue, Resistance, Ambulation (slow walking speed), Illness and Loss of Weight (5% or more in the previous year) and can be derived from pre-collected patient data (74, 77). It has been validated to predict poor outcomes such as disability and mortality, when disability is excluded from the original cohort (74). A treatment algorithm has been developed utilising its five components (78). FRAIL has been recommended in Australia as a screen for all older persons, where it can act as a “canary in the coal mine” (79); the Australian Government’s MyAgedCare website (the main point of entry into the aged care system), details information for older adults and aged care providers on how to use the FRAIL scale. The FRAIL scale is also predictive of physical limitation and mortality to a similar extent as the frailty phenotype of Fried and colleagues (80). The EFS includes nine components: functional limitation, self-reported health, general health status, cognition, social support, mood, functional performance, polypharmacy and continence (75), and is most commonly used in the hospital setting (81). It is also suitable for the community setting (82).

#### The evidence-base for frailty screening

Many frailty screening instruments are well established as predictors of adverse outcomes such as mortality, functional decline and long hospital stays (3, 73, 83–87). However, only low-certainty evidence has found that frailty screening is effective at informing treatment decisions and recovery expectations in a specialist setting (88). Indeed, the evidence-base showing that frailty screening leads to improved management of older people with frailty is lacking. This may be explained in instruments such as CFS by its focus on clinical diagnoses of disease and disability and, therefore, there is limited overlap with clinical frailty. In settings such as acute unplanned care, screening may actually have no influence on clinical decision-making regarding patient care and management (63, 89, 90).

Overall, there is a lack of current evidence to support systematic screening of frailty for all older adults, at least according to a 2018 comprehensive review (91). Many frailty screening instruments are also not validated for use in low-middle income countries (LMICs) based on a systematic review by Gray and colleagues (2016) (72).

#### Who should conduct frailty screening?

A range of health practitioners may perform frailty screening, including geriatricians, PCPs, nurses, medical specialists and allied health professionals. It is important that health practitioners receive appropriate training on frailty screening. Importantly, some members of the patient consumer group suggested that older adults could also become empowered to identify frailty in themselves.

#### Barriers to frailty screening

There are number of significant barriers to the implementation of frailty screening programmes, including a lack of public awareness of frailty, difficulties for older persons to access and afford primary care services, the acceptability of screening to older adults, and a lack of clarity regarding which treatment pathways should follow screening/diagnosis (70, 91). Primary care practices also face difficulties when adding an additional component to patient consultation visits.

#### Is frailty screening cost effective?

Due to the very low certainty of evidence on the cost-effectiveness of frailty screening (92), it is unclear whether the benefits of frailty screening outweigh its cost. Evidence to support the cost-effectiveness of frailty screening comes predominantly from a recent clinical trial by Bleijenberg and colleagues (93) which reported that proactive screening for frailty in primary care was associated with a high probability of cost-effectiveness (85). However, a recent meta-analysis of the Dutch proactive approaches (which also included data from the study by Bleijenberg and colleagues) highlighted that frailty screening is not likely to be cost-effective (86).

## Physical Frailty Assessment

### Recommendation 2: Clinical assessment of frailty should be performed for all older adults screening as positive for frailty or pre-frailty (Strong recommendation; low certainty of evidence)

The task force strongly agreed that the clinical assessment of frailty should be performed for all older adults screened as positive for frailty or pre-frailty (task force agreement with recommendation: 94.7%). This recommendation was universally supported by the healthcare provider reviewing groups and the two patient consumer groups.

#### Assessment of physical frailty

Frailty can be clinically assessed using various criteria. The ICFSR recommended standard for clinical frailty assessment is the highly validated physical frailty phenotype, developed by Fried and colleagues in 2001 (37). An older person is classified as frail when three or more of these five components are present; pre-frailty is classified when one or two components exist (37). Moderate-certainty evidence has demonstrated that frailty assessment using Fried’s frailty phenotype is beneficial for older people with frailty.

It is important clinically to differentiate frailty from multi-morbidity and disability because management of these conditions differs. A Comprehensive Geriatric Assessment (CGA) (68) is too complex to be used as a frailty assessment tool and can miss frailty as it was originally designed to assess disability (and contributing medical conditions) in older adults before the concept of frailty existed clinically (95). Thus, CGA tends to be more disability- rather than frailty-centric. In addition, the quality of a CGA assessment depends on the time and quality spent on its application, and understandably there is likely to be much variation in quality and content. Further to this, CGA assessment can be costly to perform and may not be feasible in low resource settings (33). Notwithstanding this, a comprehensive management plan for older adults can be informed by a CGA (96), and an adapted CGA (68, 97) may be performed to identify any underlying causes of frailty.

#### Who should conduct frailty assessment?

Frailty assessment should be performed by a health practitioner who has had specific training in frailty assessment. This assessment is not limited to geriatricians, but also includes other medical specialists, PCPs, and allied health professionals who have undertaken geriatric training.

## Development of a Comprehensive Management Plan

### Recommendation 3: A comprehensive care plan for frailty should systematically address: polypharmacy, the management of sarcopenia, treatable causes of weight loss, and the causes of exhaustion (depression, anaemia, hypotension, hypothyroidism, and vitamin B12 deficiency) (Strong recommendation; very low certainty of evidence)

There was strong agreement by the ICFSR task force that a treatment/comprehensive care plan be implemented for all older adults with frailty, keeping in line with an older person’s preferences, goals and level of frailty (task force agreement with recommendation: 89.5%). This care plan should include the treatment of sarcopenia, polypharmacy, exhaustion causes (depression, anaemia, hypotension, hypothyroidism, and B12 deficiency), and treatable causes of weight loss/undernutrition (5, 33, 98). Fatigue is also the result of various co-morbidities such as cardiac failure, and the task force suggest that a clinical evaluation be performed to exclude other causes of fatigue. The external healthcare provider reviewing groups also strongly agreed with this recommendation.

#### The evidence-base for the development of a comprehensive care plan

Genuine evidence on the effectiveness of a comprehensive management plan for frailty is emerging. An evaluation of the evidence-base found that a very low certainty of evidence existed regarding the effectiveness of individually tailored care plans for older adults with frailty. This evaluation was based on a recent systematic review (57), which covered three relevant clinical trials (99–101). Importantly, an individualised care and support plan to manage frailty in older adults is endorsed in a recent report by the British Geriatrics Society (BGS), Age UK and the Royal College of General Practitioners (RCGP) (68).

#### Treatment of Sarcopenia

Given the close link between reduced muscle strength and frailty, it is recommended that strategies for the management of sarcopenia are also adopted for older adults with frailty. The recent ICFSR guidelines for sarcopenia discuss specific strategies to improve aspects of sarcopenia (muscle strength, function, and muscle mass) (49). An additional benefit of focusing on the treatment of sarcopenia is that an International Classification of Diseases, Tenth Revision, Clinical Modification (ICD-10-CM) code has been recently awarded to sarcopenia (102, 103), which may be beneficial in terms of claiming for healthcare reimbursement, depending on the country.

#### Exhaustion causes

Older adults with either frailty or pre-frailty should be assessed for fatigue causes. A recent large-scale study from both Dutch and Italian datasets (the Longitudinal Ageing Study of Amsterdam (LASA) and Invecchiare in Chianti (InCHIANTI) respectively) reported that exhaustion (fatigue) was the first frailty symptom to manifest in older adults (38). Major causal factors for exhaustion include: depression (102, 104); sleep apnea (105, 106); vitamin B12 deficiency (102); hypothyroidism (104, 105); anemia (107, 108); and hypotension (109).

#### Avoidance of medication-related harm by reducing polypharmacy

A 2019 meta-analysis of 37 studies by Palmer and colleagues reported that 59% of older people with frailty are medicated with five or more medications (polypharmacy) (110). Medication management is recommended as part of a comprehensive management plan for frailty by the ICFSR task force. Appropriate de-prescription (medication withdrawal) to target polypharmacy is also recommended. A major point of contention with regards to de-prescribing in older people with frailty is the disagreement between pharmacists and clinicians regarding which medications to de-prescribe and how many should be de-prescribed (111). It is therefore recommended by the ICFSR task force that de-prescribing for older adults with frailty should occur using standard guidelines such as from the Screening Tool of Older Person’s Prescriptions (STOPP) criteria (112–114) or Beers Criteria (116). The recent Asia Pacific clinical practice guidelines for frailty (33) and the best practice guideline report for the management of frailty (68) also support de-prescribing. Recently developed, although not yet externally validated, is a frailty-specific STOPP recommendation for use by clinicians (termed STOPPFrail), which outlines 27 criteria which of potentially inappropriate medications for older adults with frailty (114).

The task force recommendation to de-prescribe is based on a limited evidence base. The majority of systematic reviews of frailty interventions in community dwelling older adults have not focused on the effectiveness of de-prescription (56, 57). There is also a lack of evidence regarding the safety and effectiveness of medications in older adults with frailty (117).

Two systematic reviews specifically on older people with frailty have reported that:

- The number of studies investigating the reduction of polypharmacy is low, and the risk-to-harm ratio is not clear (118).
- A systematic mapping review of frailty interventions in hospital emergency departments (EDs) by Preston et al. (63) reported that there was some evidence, albeit of very low certainty, that reviewing medications using implicit criteria reduced ED readmissions;

Targeting polypharmacy for older people with frailty has been found to have no significant impact on mortality or other adverse outcomes, at least based on results from a recent RCT on de-prescribing in older Australians living in residential aged care facilities (RACFs) (n = 47 intervention; n = 48 control) (119). In this study, all RACF residents over 65 years were considered as frail.

#### Other strategies

All older persons with frailty should be assessed for visual and hearing difficulties and these should be corrected when present. Those who are at risk of falling should be checked for orthostatic hypotension and syncope (120).

### Recommendation 4: Where appropriate, persons with advanced (severe) frailty should be referred to a geriatrician (CBR; no data)

The task force unanimously agreed that where appropriate, older persons with severe frailty should be referred to a geriatrician (task force agreement with recommendation: 100%). This recommendation reflects the expertise of geriatricians in managing more complex cases of frailty (121). There was support for this recommendation by the healthcare provider reviewing groups, although it was highlighted that in a rural area, geriatricians were not locally available and there were long drives/wait lists for initial consultations. In such cases, PCP management of frailty is required.

## Physical Activity

### Recommendation 5: Older people with frailty should be offered a multi-component physical activity programme (or those with pre-frailty as a preventative component) (Strong recommendation; moderate certainty of evidence)

There was a strong agreement from the task force that multicomponent physical activity should be recommended for all older adults with frailty (or pre-frailty as a preventative component) (task force agreement with recommendation: 94.7%). The healthcare provider reviewing groups completely agreed with this recommendation, suggesting that all physical activity programmes be made available by a referral process. Similarly, there was unanimous agreement amongst the patient consumer groups that physical activity was the most feasible way to prevent and treat frailty.

#### The evidence base for a multi-component physical activity programme to manage frailty

Using GRADE methodology, very low certainty evidence revealed the multicomponent physical activity programmes (combining resistance-based training with aerobic and balance training) were effective at managing frailty in older adults. Recent systematic reviews which only include trials of frailty treatment (and not combined with frailty prevention trials) have reported that multicomponent training improved the outcomes of: muscle strength, balance, disability and falls in older adults with frailty (59–61). To date, there is insufficient evidence that frailty or functional decline can be improved with multicomponent programs (59–61), predominantly because very few trials actually measure these two variables at both baseline and follow-up.

There was insufficient evidence to identify the optimal frequency, intensity, time and type (FITT) of physical activity required to treat/manage frailty. Similarly, there was insufficient literature to determine the exact combination of training modes (aerobic, resistance and balance training) most effective for frailty management. Notwithstanding this, it is understood that for physical activity programs to be effective for those with frailty, a minimal level of intensity and an adequate program timespan are needed (122). Group physical activity sessions were more likely to be successful in improving frailty than individual sessions according to a recent systematic review (57), although this premise is based on only two trials which did not use qualified trainers.

Systematic reviews of physical activity for the treatment of frailty have specifically reported that:

- Among older adults with pre-frailty, those who participated in group physical activity programs improved in physical functioning (SMD = 0.37, 95% CI 0.07 to 0.68; 3 studies) (56);
- Physical activity training improved Timed Up and Go (TUG) speed in older adults with frailty in four out of five trials, walking speed in two out of five trials, and balance in one out of three trials (59). The authors concluded that multicomponent programs incorporating resistance training were most likely to improve functional capacity in those with frailty, although they were unable to establish the optimal program type;
- The effect of physical activity in older adults with frailty was significant in improving normal gait speed (SMD = 0.07m/s, 95% CI 0.02 to 0.14) and the Short Physical Performance Battery (SPPB) (SMD = 2.18, 95% CI 1.56 to 2.80); there was no significant effect of physical activity on endurance, balance or ADL functional mobility (60). Due to a scant evidence base, the authors were unable to identify which training mode was most effective at improving physical functioning for those with frailty. A limitation of this review was that frailty was not defined using an objective screening/diagnosis instrument. Rather, frailty was identified according to ADL limitation/lowered physical functioning;
- Among older adults with frailty, physical activity intervention led to improvements in gait ability in six out of eleven trials, balance in seven out of ten trials, muscle strength in nine out of thirteen studies, and a reduction in the incidence of falls in seven out of ten trials (61). The authors concluded that a multicomponent physical activity intervention program incorporating resistance, endurance and balance training was likely to be the best strategy to improve physical outcomes in older adults with frailty. A limitation of this review was that authors included all studies which defined their participants as “physically frail”, which included frailty defined by the physical domains of Fried’s frailty phenotype, and persons aged 70 years and over with functional decline/physical performance impairments. That is, included studies did not define solely define frailty using an objective frailty instrument.
- In community-dwelling older adults with physical frailty (defined by the frailty phenotype criteria of Fried and colleagues), there was very low certainty evidence that physical activity improved muscle strength and physical performance compared with placebo (systematic overview of the literature by Lozano-Montoya et al. with evaluation using GRADE criteria) (58). Only one relevant RCT was identified in this review (123).

There are additional systematic reviews covering the effects of physical activity for older persons with frailty, although these are limited by their merging of frailty prevention studies (no persons with frailty or pre-frailty at baseline) with frailty intervention trials (57), or by including studies defining frailty using outdated definitions such as “having multiple morbidities”, “resident of an aged care facility”, “dependent in ADL” or simply “aged 65 years and over” (124, 125).

#### Patient consumer feedback regarding physical activity to manage frailty

Members of the patient consumer group advised that they would need guidance and support to undertake a physical activity program, with many preferring group-based classes. The majority of patient consumer group members also agreed that it would be very difficult to accomplish a physical activity program on their own. The importance of increasing physical activity and reducing sedentariness was also highlighted by members of the patient consumer group.

### Recommendation 6: Health practitioners are strongly encouraged to refer older people with frailty to physical activity programs with a progressive, resistance-training component (Strong recommendation; moderate certainty of evidence)

First-line therapy for the management of frailty should include a multi-component physical activity program with a resistance-based training component (task force agreement with recommendation: 94.7%). There were two main reasons as to this strong recommendation by the task force: (i) the strong dose-response relationship found in clinical trials of resistance training in older adults with frailty; and (ii) the agreement by both patient consumer groups as to the importance of resistance training.

#### The evidence base for resistance-based training

Moderate-certainty of evidence showed that older adults with frailty improve in their frailty status and physical performance when undergoing either progressive resistance-based training or a multicomponent physical activity program containing resistance-based training (57). Resistance-based training includes any physical activity which uses external resistance (such as from dumbbells, machine-based weight training, hydraulic resistance (water-based resistance training) elastic therapy bands, and body weight) to produce skeletal muscle contractions at levels higher than during routine activity (49).

## Nutrition and Oral Health

### Recommendation 7: Protein/caloric supplementation can be considered for persons with frailty when weight loss or undernutrition has been diagnosed (Conditional recommendation; very low certainty of evidence).

Recent systematic reviews indicate that in observational studies, undernutrition in community-dwelling older adults is closely associated with frailty (126, 127). However, when intervention trials are considered, only very low certainty evidence supports protein/caloric supplementation for older adults with frailty (58). Indeed, the benefits of protein/caloric supplementation are debatable if no weight loss, undernutrition or sarcopenia are present. The task force therefore recommend protein and/or caloric supplementation in older adults with frailty when weight loss is present or undernutrition has been diagnosed (task force agreement with recommendation: 78.9%). Both healthcare provider reviewing groups generally agreed with this statement, as did the majority of consumer group participants (see Appendix 1.2).

Also suggested by the task force was that a formal diagnosis of undernutrition be obtained as a basis to the recommendation of nutritional supplementation (128).

#### The evidence-base for protein/caloric supplementation

The evidence-base on nutritional supplementation is heterogeneous in terms of study outcomes and firm conclusions cannot be inferred. Systematic reviews of protein and/or caloric supplementation have reported the following:

- In community-dwelling older adults with physical frailty (defined by the frailty phenotype criteria of Fried and colleagues), it was uncertain whether phospholipid supplementation improved muscle strength or physical performance compared with placebo; evaluation with GRADE criteria showed a very low certainty of evidence for all outcomes, with a very serious risk of bias and serious issues with both imprecision and indirectness (applicability/generalisability) (58);
- Among older adults with frailty, protein-energy/protein supplementation led to increases in physical performance and strength (gait/leg strength) with a moderate-certainty of evidence (evaluated using GRADE criteria), as well as improvements in gait speed and frailty with a low-certainty of evidence (57). However, this result was deduced from only three small-scale (n < 200) clinical trials with frailty/physical performance as outcomes measures (123, 129, 130). These trials had selection biases (allocation concealment issues and lack of randomisation), performance biases (participants/study personnel not properly blinded to the study protocol), and attrition bias resulting in incomplete outcome data (57);
- When studies of older adults with pre-frailty were examined in the meta-analysis by Frost and colleagues (56), no studies of protein/caloric supplementation were identified.

#### Dietary Food Quality

Overall dietary quality may influence the progression of frailty in older adults (53, 54, 131). For instance, low-level evidence from prospective cohort studies has demonstrated that a traditional Mediterranean diet can reduce frailty risk in community-dwelling older persons (132–134). These findings were supported by a 2018 meta-analysis which reported that good adherence to a Mediterranean diet significantly reduced incident frailty [OR (95% CI) = 0-.47-0.82] (135). Lack of dietary nutrient quality may also accelerate the progression of frailty, with a recent systematic review reporting that micronutrients such as folate, β-carotene, and vitamins A, C and E associate with the development of frailty (136).

However, studies of dietary quality (including micronutrient intake) have focused primarily on the prevention of frailty development. What is not known is the effect of dietary quality for the treatment/management of already established frailty. Clinical trials involving older adults with frailty are therefore needed to form an evidence-base regarding the benefits of dietary quality for the management of frailty in older adults.

#### Treatable causes of weight loss in older adults

Treatable causes of weight loss can be identified by the MEALS-ON-WHEELS mnemonic (137, 138):

- **M**edications
- **E**motional (depression)
- **A**lcoholism, anorexia tardive, abuse (elder)
- **L**ate life paranoia
- **S**wallowing problems
- **O**ral problems
- **N**osocomial infections, no money (poverty)
- **W**andering/dementia
- **H**yperthyroidism, hypercalcemia, hypoadrenalism
- **E**nteric problems (malabsorption)
- **E**ating problems (eg, tremor)
- **L**ow salt, low cholesterol diet
- **S**hopping and meal preparation problems, stones (cholecystitis)

#### Patient consumer group feedback regarding nutritional supplementation to manage/treat frailty

No members of the Spanish patient consumer group considered that supplements would be needed to treat frailty, with participants preferring to complement physical activity with a good, healthy diet, especially a Mediterranean diet. For the USA patient consumer group, the majority of members suggested that they would not use nutritional supplementation to manage frailty, although a few members were unsure and suggested that supplements could perhaps only be prescribed if diets were deficient.

### Recommendation 8: Health practitioners may offer nutritional/protein supplementation paired with physical activity prescription (Conditional recommendation; low certainty of evidence)

Older people with frailty can be offered nutritional/protein supplementation paired with a physical activity program (task force agreement with recommendation: 89.5%). Low certainty evidence revealed that physical activity combined with nutritional intervention is effective at improving frailty, gait speed, grip strength and physical performance in older adults with frailty and/or pre-frailty (56, 57, 129, 139). The majority of this evidence is based on one small-scale RCT (n = 131) of older Japanese women with frailty (123) which found that supplementation with milk fat globule membrane (MFGM) combined with a 60 min physical activity class (twice per week for three months) reduced exhaustion and improved walking speed, although did not improve skeletal muscle mass or strength. Whilst there are other trials purported to improve outcomes for frailty by combining physical activity/nutritional supplementation (57), these studies are of older adults receiving care services (140), which are more of a population with disability, and not necessarily frail.

It is believed that nutritional intervention has an additive effect to the benefits of physical activity training, and vice versa (54, 56). Notwithstanding this, current clinical trials combining physical activity and nutritional supplementation tend to have high biases (56).

### Recommendation 9: Advise older adults with frailty about the importance of oral health (CBR; no data)

The task force suggests that older adults with frailty be advised about the importance of oral health. This advice may be incorporated as part of routine medical appointments, and can include information on oral and denture hygiene (141). Referral to oral health specialists is also advisable in some instances (141).

#### The evidence-base for oral health

Systematic review evidence indicates that oral health is associated with frailty, although the majority of research on the topic is from cross-sectional studies (142, 143). Older adults with frailty are more likely to have edentulism (reduced tooth number) and lower occlusal force (bite strength) (144). Recent longitudinal studies from Japan have reported that older adults with lower occlusal force/reduced chewing ability are significantly more likely to develop frailty (145, 146). Similarly, a study of British community-dwelling older people found that incident frailty was higher in older adults with edentulism, oral health problems or symptoms of dry mouth (147). However, there exists a shortage of clinical trials investigating the impact that improved oral health has for older persons with frailty.

## Pharmacological Intervention

### Recommendation 10: Pharmacological treatment as presently available is not recommended therapy for the treatment of frailty (CBR; very low certainty of evidence)

The task force does not recommend presently available pharmacological treatment for the management of frailty (task force agreement with recommendation: 89.5%). This statement was also supported by both healthcare provider reviewing groups.

#### The evidence-base behind pharmacological intervention for frailty

Insufficient evidence exists regarding the effectiveness of pharmacological interventions for older adults with frailty. It is therefore not possible to evaluate whether the benefits of pharmacological intervention outweighs the risk of adverse outcomes such as unwanted side effects and patient burden. Notwithstanding this, several recent studies are paving the way forwards with regards to advancing drug development for the treatment of frailty (148). It has been proposed that pharmacological-based strategies may, in time, be beneficial to those older adults with frailty, particularly for those with co-morbid conditions which exacerbate frailty (148). However, at this point in time, both the European Medicines Agency (EMA) and the Food and Drug Administration (FDA) do not consider frailty to be an acceptable condition for a medication to be approved.

There exists many challenges for pharmacological trials for the management of frailty, including the lack of a standard measurement for frailty, and defining exactly who will be in clinical trials given the different conceptual understandings of frailty (148, 149). Perhaps most notable is that older adults with frailty may have co-morbid conditions, and clinicians are advised to use their own expert clinical judgement regarding the management of these conditions — noting that these conditions may indeed warrant specific pharmacological treatment/management regimes.

## Additional Therapies and Treatments

### Recommendation 11: Vitamin D supplementation is not systematically recommended for the treatment of frailty unless vitamin D deficiency is present (CBR; very low certainty of evidence)

The task force does not recommend Vitamin D supplementation for the treatment of frailty unless there is a clear deficit (task force agreement with recommendation: 89.5%). This decision is based on disagreement over whether/how vitamin D supplementation should be prescribed for older adults with frailty (57, 150, 151). The healthcare provider reviewing groups also did not agree with Vitamin D supplementation.

#### The evidence-base for Vitamin D supplementation for older people with frailty

Frailty is associated with low levels of vitamin D in the majority of epidemiological studies (152). Recent meta-analysis evidence also suggests that 25-hydroxyvitamin D (25OHD) serum levels associate with frailty in a dose-response manner (153). However, insufficient evidence exists regarding the effectiveness of vitamin D supplementation for older adults with frailty. Indeed, there exists a distinct shortage of clinical trials which focus exclusively on frailty. Most vitamin D trials have targeted the generic population of older adults, with several systematic reviews on the topic reporting mixed results (154–158). Furthermore, there is moderate certainty evidence that vitamin D supplementation decreases fall rate but likely does not influence falls risk, at least according to a recent Cochrane review of older adults in hospital or aged care facilities (159). Notably, dosing of vitamin D may be relevant for fall prevention among frail older adults. Several double-blind RCTs comparing vitamin D with or without calcium against placebo among frail older adults reduced the risk of falling with daily applications of 700 to 1000 IU vitamin D (160). However, several trials testing large bolus applications (monthly 60’000 IU (161) to 100’000 IU (162) of vitamin D or annual dosing of 300’000 IU (163) to 500’000 IU (164)) increased fall risk among frail older adults. In terms of improvements in functional outcomes, the evidence-base is inconsistent (155–157). Further research is needed on the role of vitamin D supplementation in frailty prevention and treatment among older adults.

### Recommendation 12: Cognitive or problem-solving therapy is not systematically recommended for the treatment of frailty (CBR; very low certainty of evidence)

The task force does not recommend cognitive or problemsolving therapy for the treatment of frailty (task force agreement with recommendation: 78.9%). Current evidence is insufficient to adequately evaluate the effectiveness of either of these therapies. Although the systematic review of Apostolo and colleagues (57) concludes that moderate-certainty of evidence shows cognitive therapy improves frailty, gait speed, knee strength and exhaustion levels, this conclusion was based on only one clinical trial which had 50 participants in the cognitive training intervention (129). Regarding problem solving therapy, only one clinical trial was identified by Apostolo and colleagues’ review (57), and although this trial improved leg extension power and some secondary outcomes among older adults with pre-frailty or frailty (combined analysis) (n=57 for the problem solving therapy arm of the study) (165), the effect was much less than combined physical activity/nutritional intervention.

### Recommendation 13: Hormone therapy is not recommended for the treatment of frailty (CBR; very low level of certainty)

Given the high degree of uncertainty regarding the effectiveness of hormone therapy (33, 57), the ICFSR does not recommend hormone therapy for the treatment of frailty (task force agreement with recommendation: 89.5%). A systematic review of hormone therapy (57) found only one small-scale RCT on the topic (166), with this trial reporting that dehydroepiandrosterone and/or atamestane therapy did not improve measurements of frailty in older men with low strength at baseline.

Importantly, whilst the task force does not recommend hormone therapy for the treatment of frailty, it recognises that older people with frailty may require hormone therapy for other medical conditions.

### Recommendation 14: All persons with frailty should be offered social support as needed to address unmet care needs and encourage adherence to the Comprehensive Management Plan (Strong recommendation; very low certainty of evidence)

The task force strongly recommends that older people with frailty should be offered social support as needed to address unmet care needs and encourage adherence to a Comprehensive Management Plan. This recommendation is based on consensus of best practice (task force agreement with recommendation: 94.7%). Both patient consumer groups agreed with this statement and flagged that those with cognitive deficits would especially need this support.

#### The evidence-base for social support provision

Social isolation is a major risk factor for the progression of frailty in older adults (167, 168). Older adults with frailty may need assistance in navigating their health care, and in performing instrumental ADLs (IADLs) such as medication management and grocery shopping. These difficulties are faced by all older adults (70), although are exacerbated with frailty (2). It is also important for healthcare providers to consider autonomy in processes of care and accessibility of healthcare services for older adults with frailty (169).

The impact on social support on outcomes in older persons with frailty is uncertain. An exploratory systematic review by Gardner et al. (62) reported that in older people with frailty/risk of frailty, the provision of practical social support showed evidence of potential effectiveness in enabling health-behaviour change compared with usual care (62). However, there was no evidence of effectiveness regarding other outcomes (health/social care use, mental health, general health, and health behaviour).

### Recommendation 15: Persons with frailty can be referred to home-based training (Conditional; very low certainty of evidence)

Home-based training may be considered for older people with frailty (task force agreement with recommendation: 94.7%). Recent systematic review evidence has reported the following:

- Providing community-dwelling older persons with frailty advice on health behavior improvement has been found to improve physical functioning (with a low certainty of evidence) based on a systematic review which included six trials of health-behaviour improvement (62). No data existed regarding health behaviour improvement from the provision of health behaviour advice;
- Modifying the home environment to facilitate health behavior change was found to improve both the physical functioning and health behaviour of older people with frailty (very low certainty of evidence) compared with based on a systematic review of five relevant trials (62);

These findings are supported by two recent small-scale feasibility trials:

- In community-dwelling persons aged 65 years and over with frailty/pre-frailty, a home-based training program delivered by trained volunteers (delivering skills in either physical activity/nutrition or social support twice weekly) improved nutritional and frailty status after 12 weeks (n = 80) compared with baseline nutrition and frailty (170);
- In community-dwelling adults aged 65 years and over with pre-frailty, a ‘HomeHealth’ trial comprising of 3–6 sessions conducted by a trained support worker (delivering skills in physical activity, behavioural change techniques, nutrition and mood) improved grip strength, capacity-adjusted life-years and reduced psychological distress compared with the control group (n = 51) (171). Other outcomes did not improve and health system/carer costs were variable.

The patient consumer groups also specifically reviewed advice on health behaviour improvement for older people with frailty, and underlined the importance of a health behaviour improvement program that would give them skills to improve self-care without needing assistance of family and/or caregivers (see Appendix 1.1 for details). The recommendation for home-based training is aligned with the WHO’s Integrated Care for Older People (ICOPE) initiative (172). The task force also suggests that home-based training be performed by trained health professionals and/or trained volunteers.

## Discussion

This report provides recommendations for the identification and management of frailty in older adults developed and endorsed by the ICFSR. These recommendations were predominantly evidence-based using the GRADE methodology, and for sections where research evidence was lacking, best-practice consensus based recommendations were formed. To ensure that older adults with frailty receive the best possible quality of care, the next challenge is for the guidelines to be incorporated into routine care.

The ICFSR task force emphasise that these guidelines should be used in conjunction with clinical judgement when developing care plans for older adults with frailty. Not all older adults with frailty will respond to all therapies and treatments, particularly those with co-existing conditions or with advanced frailty. Each older person will have their own unique needs, and principles of personalised medicine should be applied when treating/managing frailty (173). Clinicians should be aware that in any intervention, benefit should outweigh potential patient harm. Whilst this recommendation is common to all medical practice, this is particularly important for older adults with frailty who unfortunately have an increased likelihood of receiving non-beneficial tests and treatments which may expose them to unnecessary burden and risk (174).

Also integral to the management of frailty is shared decision making. Shared decision making involves joint consultation on decisions between health care providers, patients and caregivers to decide upon the best care strategies, and in turn, promotes person-centred care. Factors important to older persons with frailty need to be incorporated into goals of care and treatment decisions, including preferences, values and individual priorities (175). The accessibility of resources and social support systems, or lack thereof, also needs to be accommodated.

These ICFSR guidelines build on the 2016 Asia-Pacific clinical practice guidelines for the management of frailty (33) by drawing on evidence from recent systematic reviews which have used the GRADE approach. The Asia-Pacific guidelines provide regional specific recommendations, including which specific cut-off points to use for anthropometric-based frailty screening tools, and best-practice interventions for regions with low resources and lack of accessibility to geriatrician-based care.

Into the future it will also be important to determine the feasibility of using the concept of frailty as a decision making tool for treatment goals for older adults with co-existing morbidities (176). In addition, from an implementation standpoint, these guidelines can be used to generate clinical care pathways that are locally adapted.

### Guideline Update

Over the next five years, the ICFSR task force will monitor the literature for any new advances in technologies or clinical treatments. The next update for the ICFSR guidelines on frailty is due in 2024, or earlier if breakthrough discoveries are made. It is suggested that guidelines for frailty identification and management be developed for those research fields with a larger evidence base- namely primary care, acute care, cardiology, rehabilitation, and oncology.

### Limitations

These ICFSR guidelines focus on management of the individual older person with frailty. They have not been designed from a public health perspective, although they have the potential for adaptation in order to inform public health policies. Important to note is that whilst GRADE guidelines were used as the crux of the recommendations, there were often sizeable gaps in the evidence-base which limited the application of the GRADE methodology. The steering committee filled these gaps using consensus-based best practice recommendations from the task force, the patient consumer groups, and the external evaluation group.

A major limitation in the formation of the guidelines was the lack of quality RCTs upon which to develop the guidelines. Although there are many clinical trials on frailty, the majority did not measure frailty using validated frailty instruments, and in those few studies that did, even fewer re-measured frailty at follow-up. Many trials also had heavy biases, especially with regards to patient selection and randomisation errors, incomplete datasets due to high attrition rates, and selective reporting (56). In addition, most trials were small scale (n < 200). An additional concern is that systematic reviews of frailty have a tendency to combine frailty prevention trials with trials for the treatment of frailty/pre-frailty (57, 124–125). Individuals with frailty may respond very differently to interventions than those without frailty. For instance, older adults with frailty have much higher attrition rates in clinical trials than their counterparts without frailty. We also highlight the lack of person-centred outcome measures used in RCTs on frailty. These guidelines have therefore sought the external opinion of older adults with frailty via both the patient and healthcare consumer groups.

## Supplementary Information

Appendix 1
