# Supplementary material for: Physical Frailty: ICFSR International Clinical Practice Guidelines for Identification and Management
Source: J Nutr Health Aging. 2019 Oct 3;23(9):771–87. doi: 10.1007/s12603-019-1273-z (PMC6800406; doi:10.1007/s12603-019-1273-z)
Supplement: Supplementary file 1 — Appendix 1 [file mmc1.docx]

**Appendix 1:**

**Appendix 1.1:** Patient consumer group feedback on health behaviour improvement.

| **Health Behaviour Improvement Strategy** | **Patient consumer group feedback** | |
| --- | --- | --- |
|  | **Madrid, Spain** | **Perry County, USA** |
| **1. Either your health provider or you ( ____) should yearly screen for the presence of frailty.** | Most members of the patient consumer group agreed with this statement, suggesting that yearly screening for the presence of frailty is both appropriate and needed so that older people “do not lose more”. Screening for was frailty was deemed important as a way to receive more care (if needed) and to be able to improve self-care. It would also be a way for healthcare professions to pay attention to frailty once identified. | All 12 consumers agreed with this strategy. |
| **2. If you screen positive for frailty, your health provider should look for and treat causes of fatigue, weight loss, loss of muscle function (sarcopenia) and polypharmacy (too many or “bad” medications).** | The patient consumer group agreed that this is needed and should be compulsory. However, they foresaw a problem in the fact that identifying and treating these causes of frailty would require time and dedication from a health care practitioner. The consumer group also highlighted that such time and dedication would be difficult due to high work burdens, and there could be delays in specialist referral for patients who needed this appointment. Whilst some members of the patient consumer group mentioned that only professionals should identify frailty, others suggested that older adults should become empowered to identify frailty/developing frailty in themselves. | All 12 consumers agreed with this strategy. |
| **3. Physical exercise, including resistance exercise, is the best way to prevent and treat frailty.** | There was absolute agreement with this statement be the patient consumer group. All members agreed that exercise increases muscle mass and function, and also suggested that increasing physical activity and reducing sedentariness be included in this statement. | All 12 agreed with this strategy, but many commented that they would need guidance and support to do so… for example, the group exercise program is fun and motivating. Most said it would be very difficult to do on their own |
| **4. There is no evidence to support use of supplement (including multivitamins) to prevent or treat frailty.** | No patient consumer group members considered the use of supplements to have any benefit, and stated that they preferred to complement physical activity with diet, especially a Mediterranean diet. | 8 consumers said they agreed. 4 were unsure and rely on a multivitamin but also commented supplements should only be prescribed if deficient. |
| **5. Persons with frailty should work with health professionals and their family to obtain social support and help with complying with preventative recommendations** | Most consumers agreed that persons should work with health professionals. Two persons stated that it is not easy to share frailty problems with their family as they did not want to feel like a burden to family members; they stated that they wanted to be in charge of their own health. In a group discussion, it was mentioned that family support should not be needed when an older person is frail, but rather when dependency or other health problems are already present. In general, most consumers thought of self-care as the “baseline” and family members should not be “bothered” at this point. For this, the consumer group underlined the importance of a health care program that would give them skills to improve self-care. | All 12 consumers agreed with this strategy. Note above comments about exercise and need for support. Especially those with cognitive deficits. |

**Appendix 1.2** Responses from the Healthcare Provider Reviewing Groups

| **Recommendation** | **Patient consumer group feedback** | |
| --- | --- | --- |
|  | **Madrid, Spain (n=15)^†^** | **Perry County, USA**^††^ |
| All persons over 65 years of age should be screened for frailty with a simple frailty screen, e.g., FRAIL, Edmonton Frailty Scale or Rockwood Clinical Frailty scale. | All bar one agree | All agree |
| Clinical assessment of frailty should be performed for all older adults screened as positive for frailty or pre-frailty. | All agree | All agree |
| The Comprehensive Geriatric Assessment is too complex and may miss early frailty as it is focused on disabilities. Where appropriate, persons with severe frailty should be referred to a geriatrician. | All bar 2 agree | Ideally, but in rural area not available or long drives and wait time to initial visit. Requires PCP to management |
| A comprehensive management plan for frailty should include management of sarcopenia; the causes of fatigue (depression, sleep apnea, hypothyroidism, B_12_ deficiency, hypotension and anemia); polypharmacy; and the treatable causes of weight loss. | All agree (14 strongly agree) | All agree: algorithm is very helpful |
| A separate multi-component exercise program should be recommended for all persons who have pre-frailty or frailty as a preventive component. | All agree (13 strongly agree). One geriatrician stated that it could be useful to specify “preventative component or treatment” | All agree: programs should be available with referral process |
| All persons with frailty should receive social support as needed to meet unmet needs and encourage adherence to the management program. | All agree (8 strongly agree) | All agree. Older patients need support especially those with frailty and dementia. |
| The use of testosterone and vitamin D is not recommended unless the person has a clear deficit. | 9 agree (4 strongly agree, 5 conditionally agree), 6 state insufficient evidence | All agree |
| Pharmacological treatment as presently available is not recommended. | 10 agree (4 strongly agree, 6 conditionally agree), 4 state insufficient evidence | All agree |
| Protein (calorie supplement) is not recommended unless weight loss or sarcopenia is present. | 10 agree (2 strongly agree, 8 conditionally agree), 5 state insufficient evidence | All agree |

†*n* = 15 health care providers (Madrid, Spain): 6 geriatricians, 3 residents in geriatric medicine, 3 nurse practitioners, 2 nurses specialized in geriatric medicine and 1 nutritionist.

Four response categories: strongly agree, conditionally agree, insufficient evidence and no recommendation

^††^ *n* = 7 health care providers (Perry County, USA): 1 PCP, 1 AFNP, 4 therapists, 1RD
